# The Effect of the Acid-Base Imbalance on the Shape and Structure of Red Blood Cells

**DOI:** 10.3390/cells13211813

**Published:** 2024-11-03

**Authors:** Snezhanna Kandrashina, Ekaterina Sherstyukova, Mikhail Shvedov, Vladimir Inozemtsev, Roman Timoshenko, Alexander Erofeev, Maxim Dokukin, Viktoria Sergunova

**Affiliations:** 1 Federal Research and Clinical Center of Intensive Care Medicine and Rehabilitology, V.A. Negovsky Research Institute of General Reanimatology, 107031 Moscow, Russia; snezhanna.lyapunova@yandex.ru (S.K.); kmanchenko@yandex.ru (E.S.); shvedovmike@bk.ru (M.S.); va.inozemcev@physics.msu.ru (V.I.); 2 Research laboratory of biophysics, National University of Science and Technology “MISIS”, 119049 Moscow, Russia; timoshenkoroma@mail.ru (R.T.); erofeev@polly.phys.msu.ru (A.E.); 3 Sarov Physics and Technology Institute, MEPhI, 607186 Sarov, Russia; safmlab@gmail.com

**Keywords:** red blood cell, pH, ROS, AFM, SICM, nanostructure, cytoskeleton, elastic modulus

## Abstract

Red blood cells respond to fluctuations in blood plasma pH by changing the rate of biochemical and physical processes that affect the specific functions of individual cells. This study aimed to analyze the effect of pH changes on red blood cell morphology and structure. The findings revealed that an increase or decrease in pH above or below the physiological level of pH 7.4 results in the transformation of discocytes into echinocytes and causes significant alterations in the membrane, including its roughness, cytoskeleton structure, and the cell’s elastic modulus. Furthermore, the study shown a strong connection between critical acidosis and alkalosis with increased intracellular reactive oxygen species production.

## 1. Introduction

Critical conditions can be associated with impaired oxygenation and rapidly induce changes in the body’s acid-base balance, coagulation system, and homeostasis, disrupting normal physiological functioning [1]. The body’s acid-base balance significantly affects erythrocytes’ functionality, structure, and hemodynamic properties. Red blood cells (RBCs) are vital in transporting oxygen and carbon dioxide between the lungs and tissue. The structure and function of erythrocytes largely depend on the stability of the environmental pH. It was found that alterations in pH levels affect the size and volume of erythrocytes [2,3], the cytoskeletal structure, the morphology, and, consequently, their ability to deform and pass through capillaries [4]. Alkalosis and acidosis represent extreme deviations in the body’s acid-base balance that can significantly disrupt homeostasis and lead to severe clinical consequences [5]. The blood pH level is susceptible to hypoxia because its parameters usually fall within a very narrow range of pH 7.35–7.45 [6,7]. In hypoxia, acidosis develops rapidly, accompanied by arterial vasodilation, reduced neuronal excitability, insulin resistance, and immunological imbalance. Similarly, the storage of erythrocyte suspensions may also lead to the development of acidosis. During the storage, changes in the metabolism of red blood cells lead to the accumulation of acidic metabolites and a shift in the pH of the environment [4,8].

Conversely, an increase in blood pH above 7.45 results in alkalosis, characterized by the accumulation of bases (alkalis) species. This condition involves a loss of hydrogen ions or an excess of bicarbonate ions and typically encompasses hypoxemic causes and pulmonary diseases [9].

Furthermore, acidosis can increase blood viscosity, whereas alkalosis may decrease it, affecting blood circulation and oxygen distribution to organs and tissues [10,11].

A reduction in blood pH level, which creates a more acidic environment, has been identified as a potential mechanism contributing to an increase in the number of reactive oxygen species (ROS). The efficacy of antioxidant systems, including enzymes such as superoxide dismutase, catalase, and glutathione peroxidase, is reduced in acidic conditions [12]. This results in the accumulation of ROS, as the body cannot neutralize them at the same speed. The rise in ROS can manifest in numerous ways at the cellular level, including the induction of oxidative stress and the damage of cellular components.

On the contrary, the alkaline environment itself does not directly contribute to the increase in ROS formation within erythrocytes. However, alterations in pH can indirectly impact metabolic processes and the functionality of antioxidant systems, which in turn can change the level of ROS [13].

The impact of pH on the cellular structure and the number of reactive oxygen species can be evaluated using scanning probe microscopy techniques. Employing atomic force microscopy (AFM), one can assess cell morphology, membrane nanostructure, and cytoskeleton parameters. Scanning ion conductance microscopy (SICM) enables the quantification of intracellular reactive oxygen species [14,15].

The cells’ physical properties are frequently employed as physiomarkers to detect abnormalities linked to the disease [16]. For example, alterations in erythrocyte morphology have been associated with changes in hemodynamic function [17]. Our study aimed to analyze the impact of pH alterations on the RBC conformation, membrane surface structure, cytoskeleton, cell elastic modulus, and cellular ROS concentration [14]. This approach facilitates a more comprehensive understanding of the mechanisms underlying functional RBC disorders in the context of acid-base imbalance.

## 2. Materials and Methods

The study was carried out in compliance with the Declaration of Helsinki and regulations of the Federal Research and Clinical Center of Intensive Care-Medicine and Rehabilitation, V.A. Negovsky Scientific Research Institute of General Reanimatology, Moscow, Russia, and according to the revised ethical guidelines of the World Medical Association’s Code of Ethics (Declaration of Helsinki).

A suspension of RBCs from nine healthy donors (six men and three women aged 26–45 years) was used for in vitro experiments. Before adding the cells to the phosphate-buffered saline (PBS) solution with a predefined pH level, the RBCs were washed three times in PBS by centrifugation at 400× *g* for 5 min. Thus, the RBCs were cleared of all leukocytes and platelets. Seventy-five microliters of RBCs was extracted from each donor’s sample and added to one milliliter of PBS with a specified pH level of 6.4, 7.4, and 8.4 (Immunotex LLC, Stavropol, Russia). The suspensions were divided into three groups and maintained at a constant temperature of 25°C for thirty minutes and seven hours, respectively. A monolayer of RBCs from each sample was prepared following the procedure described in reference [14].

### 2.1. Analysis of RBC Morphology

An atomic force microscopes NTEGRA Prima and NTEGRA BIO (NT-MDT SI, Russia) were used to capture images of cells. The surface morphology of erythrocytes was imaged in the air using NSG01 series probes (NT-MDT SI, Russia) with a typical radius of 10 nm and a spring constant of 5 N/m. All surface maps were acquired in the tapping mode [18] at a lateral velocity of 0.3–0.9 Hz and a resolution of 512 × 512 (standard resolution) and 1024 × 1024 (high resolution). A total of 1500 cell maps were collected. All acquired data were analyzed using the FemtoScan Online software, version 2.3.239 (5.2) (Advanced Technologies Center, Moscow, Russia, www.nanoscopy.ru (accessed on 7 August 2024)) [19]. The software enables the detection and quantification of minor structural alterations at varying scales. Two-dimensional Fourier transform was employed to deconvolve AFM surface images into maps of differing spatial resolutions, categorized as either “low” (waviness—h_1_, L_1_) or “high” (roughness—h_2_, L_2_) frequencies. Briefly, this method eliminates the distortion caused by the probe shape and allows the evaluation of different cell surface parameters by breaking the image into high and low spatial frequencies [14,20]. Subsequently, the resulting images were classified based on the generally accepted classification [21].

### 2.2. Sample Preparation for the Cytoskeleton Imaging

For sample preparation, 100 µL of RBCs was added to 500 µL of hypotonic solution (1 part 0.9% NaCl and 9 parts distilled water). Subsequently, the suspension was centrifugated at 400× *g* for five minutes without a break using a Universal 320 centrifuge (Andreas Hettich GmbH & Co., KG, Tuttlingen, Germany).

There are techniques that employ higher centrifugation speeds for the extraction of RBC ghosts [22]. It is important to note, however, that the approach used in this study does not involve the use of high-speed centrifugation or additional chemical agents (neither glutaraldehyde nor triton) for the preparation of ghosts. This allows us to avoid any additional influence on the protein–lipid structures of the RBC’s membrane. Proposed technique yields the highest quality RBC ghosts applicable for AFM imaging, allowing the collection of AFM maps with a minimal number of artifacts. Given the fragility of ghosts, any additional impact can lead to disruption of the ghost structure and the appearance of imaging artefacts [4,14].

The supernatant was then removed, leaving 75 µL of the RBCs. In the following step, 300 microliters of distilled water was added to 75 microliters of the precipitate to promote hemolysis. The resultant suspension was stirred in a Bio RS-24 mini-rotor (SIA BIOSAN, Riga, Latvia) at 8 rpm for 5 min and then kept at 4 °C for 30 min, followed by an additional 10 min at room temperature.

Subsequently, the samples were centrifuged at 400× *g* for five minutes. The obtained supernatant was removed, leaving 75 µL of ghosts in the tube. The pellet was then mixed, and 9 µL was deposited onto a microscope slide. A monolayer of ghosts was prepared using the V-Sampler (Vision, Vienna, Austria).

### 2.3. Young’s Modulus Measurement

The changes in the elastic modulus of erythrocytes were quantified using analysis of the AFM force–indentation curves. Nanosensors special development SD-R150-T3L450B-10 probes (Nanosensors, Neuchâtel, Switzerland), with a probe radius of 150 nm and spring constant of 1 N/m, were used. Young’s modulus was calculated using the SPM Nova 3.5 software (NT-MDT SI, Moscow, Russia). Over 100 cells were examined for each sample (>600 cells in total).

### 2.4. Spectrophotometry

The absorption spectra of the RBC suspension (D(λ)) were measured using the Unico 2800 spectrophotometer (United Products & Instruments, Dayton, OH, USA). The wavelength range of 500–700 nm, which corresponds to the absorption region for the hemoglobin components, was selected for the analysis. The calibration was performed on a phosphate-buffered saline solution with a specified pH value for each experiment. To acquire the absorption spectra of the erythrocyte suspension, 3 µL of RBCs was added to 3 mL of PBS solution with a pH of 6.4, 7.4, and 8.4. All measurements were conducted using a quartz cuvette. The relative concentration of oxyhemoglobin (HbO₂), deoxyhemoglobin (Hb), and methemoglobin (MetHb) was then calculated. To estimate the concentration of the hemoglobin derivatives, the collected spectra were fitted with Equation (1) using the Origin Pro 2019 software (OriginLab Corporation, Northampton, MA, USA).

(1) Dλ=ɛHbO2CHbO2L+ɛHbCHbL+ɛMetHbCMetHbL+M+Sλ4.

Here, (${ɛ}_{{HbO}_{2}},\;{ɛ}_{Hb},\;{ɛ}_{MetHb}$) represent molar absorption coefficients, L is the geometrical light path, M is the background level, and S is the Rayleigh scattering coefficient. The absorption coefficients employed in this analysis were sourced from [23]. As the erythrocytes were measured in PBS, the impact of scattering processes, which vary across the spectra, was taken into account. This resulted in estimating the respective concentrations of the hemoglobin derivatives ($C_{{HbO}_{2}}$, $C_{Hb}$, $C_{MetHb}$).

### 2.5. The Amperometric Detection of ROS

The intracellular ROS level was quantified by evaluating the maximum ion current from a single erythrocyte. The platinum-based nanoelectrodes, operating at a potential of +800 mV relative to Ag/AgCl, were utilized. This current is directly proportional to the amount of intracellular ROS entering the reaction ${2H}_{2}O_{2}={2H}_{2}O+O_{2}$.

In order to prepare Pt-based nanoelectrodes, disk-shaped carbon nanoelectrodes, isolated within quartz (ICAPPIC Limited, United Kingdom), with diameters of 60–100 nm were used. Prior to the deposition of platinum, the carbon nanoelectrodes were etched in a solution comprising 0.1 M NaOH and 10 mM KCl for 30–40 cycles, each lasting 10 s, with an initial potential of 0 mV and a final potential of +2200 mV. The platinum was deposited by cycling from 0 to 800 mV at a scan rate of 200 mV/s for 5 cycles in a 2 mM H_2_PtCl_6_ solution in 0.1 M hydrochloric acid. The fabrication process and the principle of amperometric ROS detection have been described in detail elsewhere [15,24].

The amperometric setup was equipped with an Axon Digidata 1550B ADC-DAC converter (Axon Instruments, San Jose, CA, USA), a MultiClamp 700B patch clamp amplifier (Molecular Devices, Wokingham, UK), a PatchStar Micromanipulator (Scientifica, Maidenhead, UK), and an inverted optical microscope Opto-Edu A16.1098 (Beijing, China). The potential difference between the Pt nanoelectrode and the reference (Ag/AgCl) electrode was recorded with the pClamp 11 software suite (Molecular Devices, Wokingham, UK).

The sample was prepared by mixing 5 μL of RBCs with 1 mL of PBS with a pH of 7.4. Subsequently, a suspension of 200 μL RBCs was seeded onto poly-L-lysine-modified 35 mm Petri dishes. The samples were stored for 40 min at 24 °C. The oxidation current of hydrogen peroxide was measured directly inside the erythrocytes. The maximum current for each measurement was used to estimate the level of ROS inside the cell. Furthermore, the results were normalized to the current at −800 mV in a 1 mM solution of ferrocene in PBS. For each sample, up to 90 cells (about 540 cells in total) were analyzed.

### 2.6. Western Blot Analysis

The sample preparation involved isolating erythrocyte ghosts, a procedure previously outlined in Materials and Methods Section 2.2. In the next step, the sample was subjected to five rounds of centrifugation at 400× *g* to ensure the complete release of hemoglobin. The protein concentration was quantified using the BCA Protein Assay Kit (Thermo Fisher Scientific, Waltham, MA, USA).

A 15% polyacrylamide gel was prepared for protein electrophoresis, and 30 µg of protein was loaded into each well. Subsequently, the gel was transferred onto a 0.45 µm nitrocellulose membrane and blocked in 5% dry milk for one hour at room temperature.

To detect actin, a primary antibody to actin (Monoclonal Anti-Actin antibody produced in mouse, 1:3000, Sigma-Aldrich, St. Louis, MO, USA) and a secondary antibody, HRP-conjugated anti-mouse IgG (1:3000, Invitrogen, Waltham, MA, USA), was utilized.

Bands were detected using SuperSignal West Pico Plus Chemiluminescent Substrate (Thermo Fisher Scientific, Waltham, MA, USA) and quantified with the imaging software on the Vilber Lourmat Fusion Solo S system (Vilber, Collégien, France).

### 2.7. Statistical Analysis

The data analysis was performed using the OriginPro 2019 software. Sample statistics were presented as the mean and standard deviation (mean ± SD). The nonparametric Mann–Whitney test was employed to assess the significance of differences between the experimental data and the reference value. A *p*-value of 0.05 was used to determine whether the observed differences were statistically significant. The Image Analysis software (NT-MDT SI, Moscow, Russia) was used to analyze the sample’s pore parameters. Pearson’s correlation coefficient was estimated.

## 3. Results

**Figure 1:**
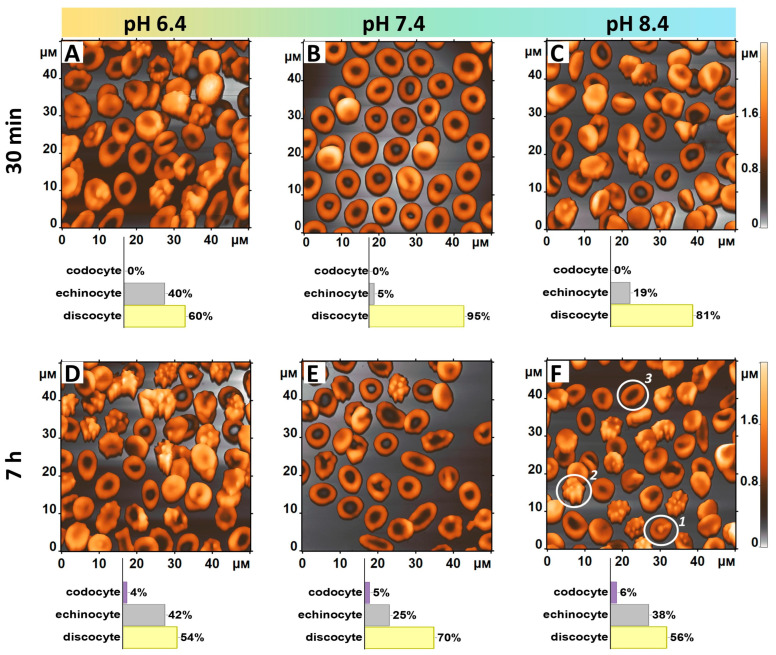
The AFM images of RBCs stored in a medium with pH levels of 6.4, 7.4, and 8.4 for 30 min (**A**–**C**) and for 7 h (**D**–**F**) are presented. The number of cells assigned to each cell type is indicated in the legend provided below each AFM map. The following shapes of RBCs are indicated in white circles in Figure (**F**): (1) codocyte, (2) echinocyte, and (3) discocyte.

**Figure 2:**
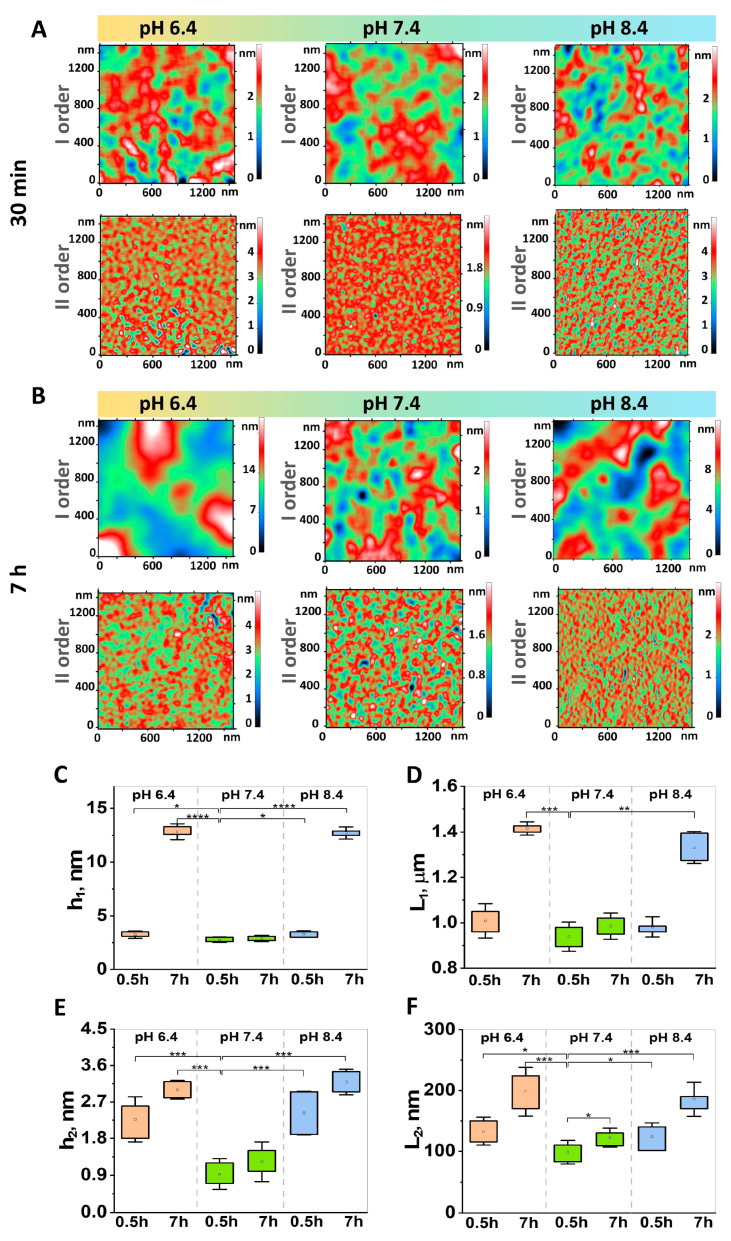
The membrane nanostructure of RBCs stored at pH 6.4, pH 7.4, and pH 8.4 for 30 min (**A**) and 7 h (**B**). Two-dimensional Fourier transform was employed to deconvolve AFM surface images into maps of differing spatial resolutions, categorized as either “low” (waviness—h_1_, L_1_) (I order) or “high” (roughness—h_2_, L_2_) (II order) frequencies. The effects of storage time on the h and L parameters are illustrated in the right-hand column (**C**–**F**). Orange color indicates pH 6.4 Green color indicates pH 7.4 Blue color indicates pH 8.4. The Mann–Whitney test was used to determine statistical significance, with * *p* < 0.05, ** *p* < 0.01, *** *p* < 0.001, and **** *p* < 0.0001 indicating a statistically significant difference.

**Figure 3:**
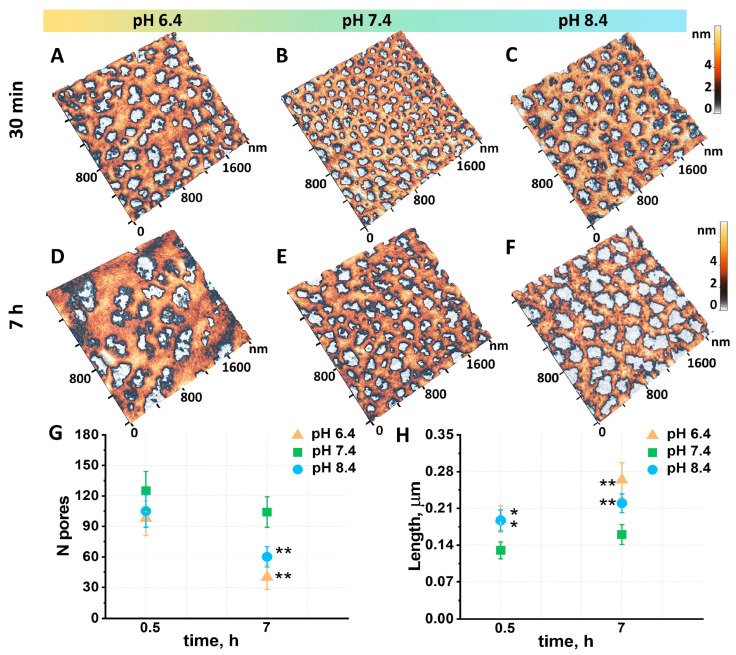
High-resolution images of the RBC cytoskeleton for samples stored at pH 6.4, pH 7.4, and pH 8.4 for 0.5 (**A**–**C**) and 7 (**D**–**F**) h. The impact of seven-hour storage on the number of pores (**G**) and pore length (**H**) is illustrated. The data are statistically significant at the * *p* < 0.05 and ** *p* < 0.01 levels, as determined by the Mann–Whitney test.

RBCs were extracted from the donor’s blood within 15 min of the blood collection. Morphometric parameters of the RBCs kept in phosphate-buffered saline at pH 7.4 for 30 min were used as a control. Morphology, nanostructure, and cytoskeleton parameters were chosen as the essential metrics for characterizing erythrocytes at different pH levels (Figure 1, Figure 2 and Figure 3).

A significant change in RBC morphology was observed following a 30 min incubation period at pH = 6.4 (Figure 1A) in comparison to the control samples stored at pH = 7.4 (Figure 1B). A notable decrease in the total number of discocytes was observed, with a reduction of 1.6 times (*p* = 0.00216). Conversely, there was an eightfold increase in the number of echinocytes (*p* = 0.00193). Furthermore, incubation in a solution at pH 8.4 (Figure 1C) also resulted in the transformation of discocytes into echinocytes. Compared to the solution with pH 7.4, the number of echinocytes increased by a factor of 3.8. The storage of the RBC’s suspension for 7 h altered the morphological cell structure in all experimental groups (see Figure 1D–F). In the control group (pH 7.4, Figure 1E), discocytes remain the predominant cell type, although their total number has decreased by 1.4 times. The number of echinocytes increased 5 times, and the codocyte cell type emerged. Keeping the cells at pH = 6.4 (Figure 1D) resulted in a notable increase in echinocytosis (to 42%) and a corresponding decrease in discocytes (to 54%). Additionally, a small number of codocytes were identified. A comparable dynamic was observed for the RBC sample stored at pH 8.4 (Figure 1F), with discocytes undergoing a notable transformation into echinocytes (38%) and codocytes (6%).

The roughness of RBC’s surface was characterized as outlined in reference [20]. Figure 2 shows the L_1_, L_2_, h_1_, and h_2_ parameters for all samples.

After a 30 minute incubation at pH 7.4, the surface parameters were L_1_ = 939 ± 64.1 nm, L_2_ = 98.5 ± 19.3 nm, h_1_ = 2.7 ± 0.2 nm, and h_2_ = 0.9 ± 0.3 nm (Figure 2C–F). Following a seven-hour storage period at the same pH of 7.4, the L_1_, h_1_, and h_2_ parameters remained unchanged. However, there was a statistically significant (*p* ≤ 0.05) increase in L_2_ by a factor of 1.3. Nevertheless, exposure to more acidic and alkaline environments with pH values of 6.4 and 8.4, respectively, led to substantial alterations in the membrane structure. The experimental data demonstrate that storage at pH 6.4 for 7 h significantly increased L_1_ (1.5 times, *p* < 0.001). Similarly, the L_2_ exhibited a 2-fold increase (*p* < 0.001). At the same time, h_1_ and h_2_ demonstrated a 5-fold and 3.3-fold increase, respectively (*p* < 0.001) (see Figure 2). The exposure of RBCs to pH 8.4 for 7 h resulted in a comparable, to that of pH 6.4, alteration in surface parameters. The value of L_1_ increased by 1.4 times (*p* < 0.01), L_2_ increased by 1.8 times (*p* < 0.001), h_1_ increased by 5 times (*p* < 0.0001), and h_2_ increased by 3.4 times (*p* < 0.001) in comparison to the control sample. These changes may indicate alterations in the RBC’s structure and functionality, with a notable impact on the nanostructure of the RBC membrane. It can be reasonably assumed that the observed transformations are primarily determined by the cell’s cytoskeleton [4].

Figure 3 shows high-resolution 2 × 2 μm^2^ AFM images of the RBC cytoskeleton for samples stored at pH 6.4, pH 7.4, and pH 8.4 for 0.5 and 7 h. The number and length of the cytoskeletal pores werecalculated using the Advance Watershed segmentation method.

RBC samples stored at pH 7.4 for 30 min (Figure 3B) had 125 ± 19 pores per 2 × 2 μm^2^. The samples stored at pH 6.4 (Figure 3A) and pH 8.4 (Figure 3C) demonstrated a statistically insignificant reduction in pore quantity. The number of pores in all samples was reduced following a seven-hour storage period. However, samples stored at pH 6.4 and pH 8.4 demonstrated a pore density that was 2.6 and 1.7 times lower, respectively, than that observed in the control samples. The length of the cytoskeletal pores, defined as the maximum possible distance between two sides of the pore, was found to be 0.13 ± 0.016 µm for the control samples (pH 7.4, 30 min). Meanwhile, the pore length for the pH 6.4 and 8.4 samples increased by 1.5 (*p* < 0.05). Following a seven-hour storage period, samples kept at pH 7.4 exhibited no significant changes in pore length. In contrast, samples stored at pH 6.4 (Figure 3D) and pH 8.4 (Figure 3F) demonstrated a notable increase in pore length, reaching 1.7 and 1.4 times their original length, respectively (*p* < 0.01). Such a cytoskeletal network rearrangement can be attributed to the disruption of cytoskeletal filaments and the clustering of protein complexes.

It is noteworthy that a robust correlation between the L_2_ parameter and the pore length was observed across all pH levels. At pH 6.4, the Pearson correlation coefficient $r_{pH\;6.4}$ was 0.86, at pH 7.4 $r_{pH\;7.4}$ = 0.89, and at pH 8.4 $r_{pH\;8.4}$ = 0.87. This correlation may be attributed to the rearrangement of the erythrocyte cytoskeleton.

**Figure 4:**
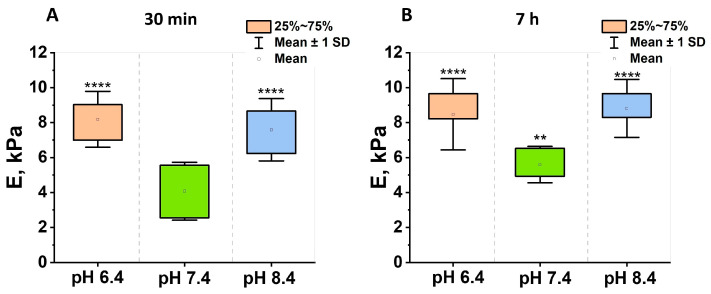
Young’s modulus (E) as a function of pH. Box plots of erythrocyte Young’s modulus in solutions of different pH held for 30 min (**A**) and 7 h (**B**). The box indicates the 75th percentile and the whiskers indicate the mean standard deviation. The square represents the mean. ** *p* < 0.01; **** *p* < 0.0001 (Mann–Whitney test).

Additional Western blot analysis (Figure S1) demonstrated the presence of actin in the samples at pH 6.4 and pH 7.4, which had been stored for 30 min. However, no actin was observed at pH 8.4. After seven hours, a distinct actin line was observed exclusively at pH 6.4, while actin traces were absent at pH 7.4 and 8.4. These results may be associated with the activity of ADF/cofilins on actin filaments, which play a critical role in the reorganization of actin filaments in response to alterations in pH [25,26]. The mechanical properties of erythrocytes were measured following a 30 min (Figure 4A) and 7 h (Figure 4B) storage period in solutions with specific pH levels (6.4, 7.4, and 8.4). Figure 4 illustrates the correlation between Young’s modulus (E) and the environmental pH. As shown in Figure 4, Young’s modulus for the sample stored for 30 min at pH 7.4 was 4.1 ± 1.6 kPa. Notably, the modulus of samples stored at pH 6.4 was approximately 2 times higher compared to the control group, with a statistically significant difference (*p* < 0.0001). Similarly, storage at pH 8.4 resulted in a 1.85 times increase in modulus relative to the control, with a statistically significant difference (*p* < 0.0001).

Following a seven-hour incubation period, the qualitative differentiation of erythrocyte mechanical properties remained similar to that observed at the initial 0.5 h time point. However, the mean Young’s modulus value slightly increased for all samples. For the sample at the physiological pH level, the modulus increased to 5.6 ± 1.0 kPa (1.2 times). The Young’s modulus of samples stored at pH 6.4 and 8.4 increased by 1.1 and 1.2 times, respectively (*p* < 0.0001). Overall, it can be seen that RBCs stored above or below the normal pH of 7.4 exhibit higher values of elastic modulus.

**Figure 5:**
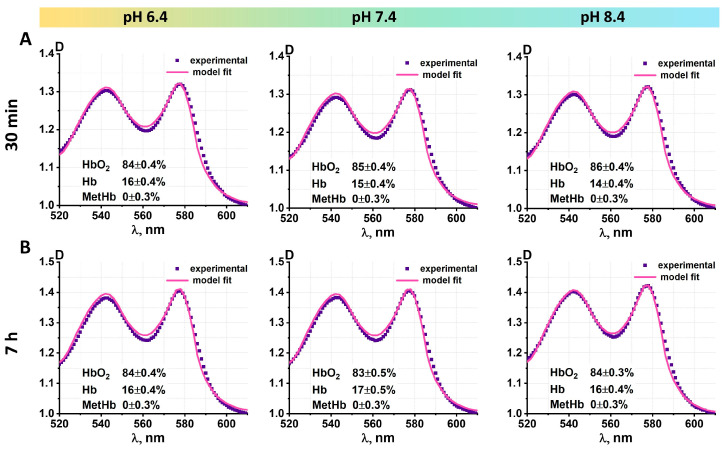
The optical spectra of erythrocyte suspensions stored in solutions with varying pH levels for 30 min (**Panel A**) and 7 h (**Panel B**) are presented. The respective concentrations of the hemoglobin derivatives were estimated based on the optical density of the solutions. All values are displayed in each graph.

This study employed spectroscopic techniques to quantify the proportion of hemoglobin derivatives in erythrocyte suspensions as a function of pH level and storage time (Figure 5).

In this experiment, we compare the effects of incubating erythrocytes in PBS solutions with varying pH levels for 30 min. The data indicated no statistically significant difference in the levels of HbO_2_, Hb, and MetHb in all samples during the 30 min incubation period. The concentration of HbO_2_ was found to be 84 ± 0.4% for a pH of 6.4, 85 ± 0.4% for a pH of 7.4, and 86 ± 0.4% for a pH of 8.4. The concentration of Hb was found to be 16 ± 0.4%, 15 ± 0.4%, and 14 ± 0.4% for pH levels of 6.4, 7.4, and 8.4, respectively. The MetHb fraction was absent in all samples.

Following a seven-hour incubation period, no changes in the percentage of hemoglobin components were observed in all samples (Figure 5B). The exposure of erythrocyte suspensions to varying pH levels did not result in any alterations to the heights or positions of the peaks observed on the absorption spectra. Characteristic HbO_2_ peaks were observed in all samples at 542 nm and 577 nm.

Changes in the pH of the medium in which erythrocytes are located cause changes in their morphology, nanostructure, cytoskeleton, and stiffness. That is, changes in the acid-base balance affect the external and internal organization of the cell but do not change the molecular structure of hemoglobin or the oxygenation properties of the cells. Therefore, the spectral properties of hemoglobin remain constant (Figure 5).

**Figure 6:**
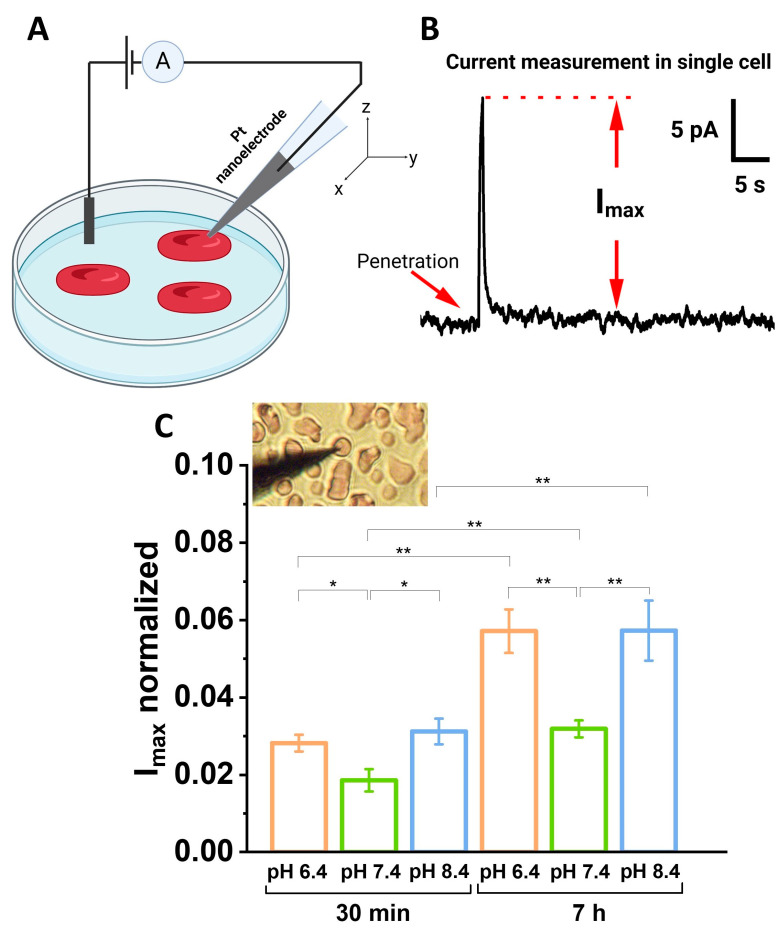
ROS measurement in a single RBC by SICM. (**A**) Experimental scheme. (**B**) Registration process of maximum current in a single RBC at a potential of +800 mV. (**C**) Comparison of normalized maximum current in RBCs during storage in pH 6.4, pH 7.4 and pH 8.4 solutions for 30 min and 7 h. The inset shows the process of approaching the Pt nanoelectrode to the single RBC. Results are shown as means ± SEM. * *p* < 0.05; ** *p* < 0.01 (one-way ANOVA).

Subsequently, we analyzed the intracellular level of ROS in an individual erythrocyte, which serves as a biomarker of acidosis or alcolosis. This is because alterations in pH can indirectly affect metabolic processes and the activity of antioxidant systems, which can alter the level of ROS [27,28,29]. The amperometric detection of ROS using Pt nanoelectrodes (Figure 6A) was used as a low-invasive technique for single-cell analysis in real-time. This method involved recording the current inside a single cell at a potential of +800 mV, at which ROS decompose (Figure 6B) [24].

The levels of ROS observed in RBCs following 30 min of incubation at pH 6.4 and pH 8.4 were found to be indistinguishable at the 95% confidence level. However, a statistically significant difference (*p* < 0.05) was observed when the pH 6.4 and pH 8.4 samples were compared with the pH 7.4 control sample (Figure 6C). Furthermore, following the long-term storage of RBCs for seven hours in pH 6.4 and pH 8.4, the level of ROS exhibited a twofold increase compared to the 30 min incubation period. It is also noteworthy that the level of ROS for the pH 7.4 sample stored for seven hours increased by 1.7 times compared to that observed after thirty minutes of incubation. These results indicate that critical acidosis and alkalosis are closely related to the induction of intracellular ROS production.

## 4. Discussion

The morphology of erythrocytes is associated with the cell phenotype and blood disorders [30,31]. In response to alterations in homeostasis or exposure to stimuli, erythrocytes may undergo transformation into echinocytes, stomatocytes, or spherocytes [32], which typically indicate specific pathological conditions [33].

The results of the in vitro experiments indicated that erythrocytes undergo morphological alterations when subjected to abnormal acid-base conditions. Following a seven-hour exposure to severe acidosis or alkalosis, the discocytes underwent a transformation into echinocytes (Figure 1). Furthermore, exposure to non-physiological conditions results in disruptive processes within RBCs, including alterations in cell surface roughness and changes to cytoskeletal parameters, such as the number and length of pores. It is important to note that the elastic modulus of erythrocytes exhibited a nearly twofold increase when incubated in both acidic and alkaline environments, compared to the physiological pH level of 7.4. The overproduction of ROS, as observed in the experiment, has the potential to affect the acid-base balance, resulting in damage to the erythrocyte membrane structure and function [29]. A strong correlation was observed between the elastic modulus and the amount of intracellular ROS production for all pH values. At pH 6.4, the Pearson correlation coefficient was found to be $r_{pH\;6.4}=0.81$, at рН 7.4 $r_{pH\;7.4}=0.91$, and at рН 8.4 $r_{pH\;8.4}=0.92$.

Significant changes in erythrocyte cytoskeleton parameters were demonstrated during 7 h of storage. At pH 6.4, a notable thickening of the cytoskeleton filaments was observed, accompanied by a twofold increase in the size of the cytoskeleton pores compared to the control sample. The number of pores at this pH was lower than at pH 7.4, indicating that the filaments were undergoing aggregation and enlargement. The shape and deformability of RBCs are supported by a planar network of short actin filament (F-actin) nodes (with a length of approximately 37 nm—15–18 subunits) that are interconnected by long spectrin strands at the inner surface of the plasma membrane [34]. The alterations in the cytoskeleton are likely associated with the fact that under acidic conditions, spectrin reaches a point of isoelectricity, which results in a reduction in electrostatic repulsion between molecules and their aggregation [35,36].

At a neutral pH of 7.4, the dimensions of cytoskeletal pores are maintained, and filaments remain in their native structure. This finding is consistent with data indicating that spectrin retains its stability and functionality at neutral pH values, where spectrin filaments are not prone to aggregation [37].

At pH 8.4, an increase in pore size is observed, yet spectrin filaments do not thicken. In an alkalosis environment, spectrin oligomers, including tetramers and higher polymers, dissociate significantly into their monomeric subunits (α- and β-subunits). This phenomenon is attributed to alterations in electrostatic interactions and increased protein charge in an alkaline condition, ultimately disrupting weak subunit interactions [38].

The cell elastic modulus also increases when the cytoskeleton morphology changes at a different pH. This may be connected with the fact that there is disruption of the filaments, the merging of small pores into larger ones, a reduction in the number of pores, and thickening or clusterization of filaments [4]. Cytoskeletal pores likely support the plasma membrane, and their size affects its mechanical properties. When they are enlarged, the cell loses its ability to optimally distribute mechanical load, which can lead to increased cell stiffness.

The blot analysis demonstrated the change in the amount of actin present in erythrocytes over thirty minutes and seven hours of incubation. Cofilin can bind to actin filaments, and its activity is subject to variation in the pH. Cofilin exhibits a greater affinity for filaments at lower pH, resulting in enhanced resistance to rupture and depolymerization. Conversely, in an alkaline environment, the binding capacity of cofilin declines, reducing the stability of actin filaments and their disassembly. This provides a potential explanation for the absence of actin at pH 8.4 after an extended period. Therefore, spectrin and actin are responsive to changes in pH, reflected in changes in the erythrocyte cytoskeletal structure.

It is notable that the alterations in morphology, roughness, and ROS production in response to pH 6.4 and pH 8.4 are similar. This may be explained by the comparable mechanisms of the cytoskeleton reaction to changes in acid-base conditions.

The present study demonstrates that extreme pH changes can significantly impact erythrocytes’ morphology and functional characteristics, including alterations in the membrane structure, cytoskeleton, and mechanical properties. Such changes could lead to significant health issues, for example, in preterm infants, where the erythrocyte membrane is prone to pathological changes with pH decrease due to hypoxia [39,40,41,42,43].

This is also important for understanding clinical acidosis while preserving blood storage. There, the pH value decreased from 7.4 to 7.0 by the 19th day and continued to drop, reaching 6.5–6.4 by the 42nd day of storage [4]. Such oxidative processes can stimulate lipid peroxidation, clustering of Band 3 protein, breakage of cytoskeletal filaments, and lead to polymerization and aggregation of protein structures within the matrix network, thereby increasing the disruption of the erythrocyte cytoskeletal nanostructure [44,45]. This is why cytoskeletal degradation processes and the increase in elastic modulus accelerate after the midpoint of the storage period when the pH drops below 6.8 [4].

When the blood pH exceeds the normal range (7.35–7.45), it can disrupt the membrane potential, leading to cell surface deformation and alterations in its morphology [17]. This suggests that erythrocytes have optimal mechanical properties and configuration at the pH level of 7.4, the natural physiological pH level. The effects of an unphysiological pH level on erythrocyte structural properties will impact the transportation of oxygen and carbon dioxide and consequently influence metabolism across the membrane. Furthermore, it could affect the viscosity of the blood itself.

## 5. Conclusions

The molecular adaptation of erythrocytes to fluctuations in the acid-base balance of blood results in notable morphological and nanostructural alterations. Under normal conditions, the blood pH is maintained within a narrow range of pH 7.35–7.45, ensuring the erythrocyte shape’s stability. However, when this equilibrium is disrupted, for instance, in the case of acidosis (a reduction in pH) or alkalosis (an increase in pH), substantial transformations of erythrocytes occur. This typically results in the transition into echinocytes, which is accompanied by alterations in the structure of the cell membrane and cytoskeleton. Atomic force microscopy is an effective technique for comprehensive analysis of such changes. AFM enables the acquisition of high-resolution images and provides insights into how pH variations influence erythrocyte conformational structure. In vitro experiments have demonstrated that deviations from the physiological pH of 7.4 result in significant changes to cell morphology, accompanied by alterations in cell surface roughness and cytoskeleton modifications. Additionally, fluctuations in acid-base equilibrium have been observed to influence the Young’s modulus of erythrocytes, resulting in a notable increase in their stiffness. These changes indicate structural rearrangements within cells in response to stress conditions caused by abnormal pH levels, which can be defined as a shift in the acidity or alkalinity of the cellular environment. Furthermore, it has been demonstrated that critical acidosis and alkalosis significantly increase the intracellular production of reactive oxygen species, which indicates cellular stress. Consequently, our results illustrate a correlation between pH alterations, erythrocyte morphology, their cytoskeleton, and ROS induction. These findings may provide insight into the advancement of novel diagnostic techniques and therapeutic strategies for treating diseases associated with acid-base imbalances and their impact on erythrocyte functionality.

## Data Availability

The datasets used and analyzed during the current study are available from the corresponding authors upon request.

## Supplementary Materials

The following supporting information can be downloaded at: https://www.mdpi.com/article/10.3390/cells13211813/s1, Figure S1 shows the Western blot analysis, which was used to reveal how the amount of actin changes during 30 min and 7 h of incubation of erythrocytes stored in PBS with different pH values.
