# Supplementary material for: The Effect of the Acid-Base Imbalance on the Shape and Structure of Red Blood Cells
Source: Cells. 2024 Nov 3;13(21):1813. doi: 10.3390/cells13211813 (PMC11545479; doi:10.3390/cells13211813)
Supplement: Supplementary file 1 [file cells-13-01813-s001.zip › cells-3253006-supplementary.pdf]

Supplementary Information for

The Effect of the Acid-Base Imbalance on the Shape and Structure of Red Blood Cells

Authors: Kandrashina Snezhanna S., Sherstyukova Ekaterina A., Shvedov Mikhail A., Inozemtsev Vladimir A., Timoshenko Roman V., Erofeev Alexander S., Dokukin Maxim E. and Sergunova Viktoria A.

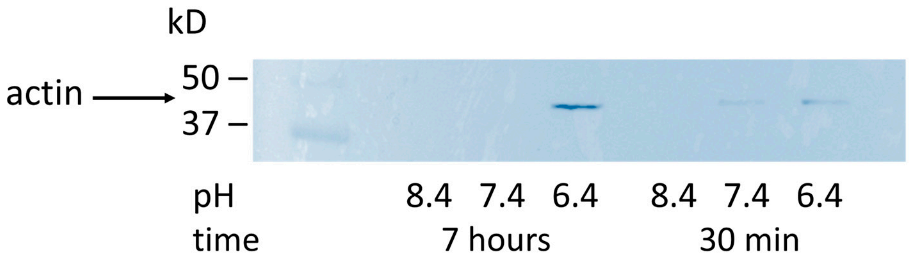

Figure S1: Western blot analysis, which was used to reveal how the amount of actin changes during 30 min and 7 hours of incubation of erythrocytes stored in PBS with different pH values.
